# Detection of *Plasmodium* using filter paper and nested PCR for patients with malaria in Sanliurfa, in Turkey

**DOI:** 10.1186/s12936-016-1334-2

**Published:** 2016-05-28

**Authors:** Nebiye Yentur Doni, Fadile Yildiz Zeyrek, Adnan Seyrek

**Affiliations:** Department of Medical Microbiology, Vocational School of Health Services, Harran University, İpekyol Avenue No: 1, 63050 Sanliurfa, Turkey; Department of Medical Microbiology, Faculty of Medicine, Harran University, Sanliurfa, Turkey; Department of Medical Microbiology, Faculty of Medicine, Fırat University, Elazig, Turkey

**Keywords:** Dried blood spot testing, Malaria, Microscopy, Nested PCR, *Plasmodium vivax*, Turkey

## Abstract

**Background:**

The objective of this study to detect *Plasmodium* and a subspecies of *Plasmodium* using filter paper in malaria endemic province, Sanliurfa, in Turkey, compare the results of nested PCR (nPCR) with microscopy for the diagnosis of malaria and present the epidemiological data of malaria.

**Methods:**

This study was carried out in malaria-endemic Sanliurfa between 2008 and 2011. Finger prick blood samples, thick and thin Giemsa-stained blood smears, were collected from 153 malaria-suspected farmworkers. The Giemsa-stained blood smears were examined microscopically. The obtained DNA products, extracted from blood-spotted filter papers or from the thick blood smears, were analysed by nPCR to amplify the 18S ssrRNA *Plasmodium* gene with genus and specific primers. The results of the microscopy were compared to the nPCR results.

**Results:**

Of the specimens, 7.2 % were determined as *Plasmodium*-positive by microscopy, whereas 9.8 % were determined as *Plasmodium*-positive by nPCR. Of the positive *Plasmodium* specimens, 93.33 % were identified as *P. vivax*. Four out of the 15 specimens that were microscopically diagnosed as negative were *Plasmodium*-positive with nPCR. When compared to the microscopy, the sensitivity, specificity, and positive predictive values of the nPCR were determined as 100, 97.2 and 73.3 %, respectively. nPCR was determined to be more sensitive and specific than microscopy.

**Conclusions:**

This study revealed that the accurate diagnosis of malaria by nPCR was compulsory in malaria-endemic Sanliurfa and nPCR should be applied routinely in laboratory studies.

## Background

Malaria is the major cause of morbidity and mortality in adults and children worldwide. According to the World Health Organization (WHO), there were an estimated 198 million cases of malaria in 2013, of which approximately 82 % were in the African Region, followed by South-East Asia (12 %), and eastern Mediterranean regions (5 %). About 8 % of the globally estimated cases are due to *Plasmodium vivax* and this ratio increases to 47 % outside the African continent [1].

Accurate diagnosis is an important tool in the fight against malaria and universal access to a parasitological test is part of the WHO objectives [1]. Microscopy depending on Giemsa-stained blood smears has been considered as the reference gold standard [2–6] for the diagnosis of malaria for more than a century. On the other hand, microscopy techniques fail to detect mixed infections, when one of the *Plasmodium* species is present at low levels (<100 parasites/mL), or modified by anti-malarial drug treatment [6, 7] and it is also a labour-intensive procedure and requires well-trained personnel [4]. However, molecular techniques have been capable for the detection and identification of malaria parasites with low and mixed parasitaemia [8]. According to the WHO, PCR was determined as more sensitive and specific than all other techniques. It does, however, require specialized and costly equipment and reagents, as well as laboratory conditions that are often not available in the field [5, 9].

The Southeastern Anatolia Region (GAP) is the most malaria-endemic region, where one of the two largest malaria epidemics of Turkey was occurred in 1994 with 84,345 cases [10]. According to Health Ministry data, it was estimated that 89 % of 36,842 malaria cases were detected in the GAP of Turkey [11].

In the GAP of Turkey, malaria transmission is seasonal, generally occurring between March and October, and shows a marked local distribution [12]. *Plasmodium vivax* is the only agent of indigenous malaria cases and only imported *Plasmodium falciparum* cases are seen in Turkey [13–16]. The GAP is one of the relatively less developed regions of the country, comprising nine administrative provinces (Adiyaman, Batman, Diyarbakır, Gaziantep, Kilis, Mardin, Siirt, Sanliurfa, and Sirnak) in the basins of the Euphrates and Tigris, and in Upper Mesopotamia. In this region, improper or excessive use of irrigation channels and deficiency in irrigation water management lead to puddles and standing water near fields. These puddles, standing water, and swamps contribute to the development of parasite larvae and breeding of *Anopheles* mosquitoes. As reported by the WHO, *Anopheles* mosquitoes breed in water and each species has its own breeding preference; for example, some prefer shallow collections of fresh water, such as puddles, rice fields, and hoof prints [17]. The GAP has been converted into an appropriate environment including temperature and climate changes for mosquito breeding and the development of parasites. Farm workers living close to puddles, standing water, and swamps have become a risk group for malaria. Many seasonal farmworkers come to Sanliurfa from different parts of Turkey to work in agriculture and many of them move from their home towns to other provinces of Turkey. The seasonal workers raised serious concerns that malaria acquired in Sanliurfa would be disseminated to other regions of Turkey. Sanliurfa is located on the board with Syria where a large influx of displaced persons from neighbouring countries.

These environmental conditions and migration from neighbouring countries result in Sanliurfa being a malaria-endemic province. Generally, the diagnosis of malaria depends on microscopical examination of thick and thin Giemsa-stained blood smears in this study area. There were no data about the diagnosis of malaria using nested PCR (nPCR) in Sanliurfa. This was the first study to compare nPCR and microscopy in Sanliurfa, in Turkey.

In light of these data, the aim of this study was to detect *Plasmodium* and a subspecies of *Plasmodium* using filter paper and compare the results of nested PCR (nPCR) with microscopy for accurate malaria diagnosis and present the epidemiological data in Sanliurfa, southeastern Turkey.

## Methods

### Study area and population

This study was conducted between 2008 and 2011 in three towns (Centre of Sanliurfa, Harran, Siverek and Akcakale) of Sanliurfa (latitude: 37.16708, North; longitude: 38.79392, East), where malaria was seen throughout the year with peaks during September to November. Average annual temperature is 19.83 °C and monthly minimal temperature −4.3 °C in January and maximal 44.2 °C in July. Average annual relative humidity is 45.27 % and rainfall is 518.91 mm [18].

After explaining the aim of the study, written informed consent was obtained from participants or their parents who work as seasonal farmworkers on agriculture of paddy, cotton, rice fields, etc., or the participants’ parents. All of the participants were asked to fill out a standardized questionnaire, which included sociodemographics and living conditions: age, gender, education, the story of malaria of the participant and his/her family in the past, history of travel abroad, presence of a chronic disease, and presence of a sewerage or stream close to their home. The main agent of the indigenous malaria transmission is due to *P. vivax*, and in recent years, foreign originated *P. falciparum* cases were frequently seen in the region [19].

The investigators visited and actively screened Akcakale, Harran, and Siverek (by house-to-house screening) with malaria experts and technicians from the Malaria Eradication Centre of Sanliurfa. In this study, a total of 153 malaria-suspected farmworkers or farmworkers’ children with at least a few malaria symptoms, including fever, headache, chills, and vomiting were determined.

### Sample collection

An aliquot of venous blood (100–200 µL) was taken by finger prick from 153 malaria-suspected farmworkers or their children, adsorbed onto Whatman^®^31ETCHR filter paper (Whatman, Piscataway, NJ), air-dried, and stored at room temperature until DNA extraction.

### Microscopic determination of the parasite, and counting the parasitemia

Thin and thick Giemsa-stained blood smears were prepared during the collection of the blood specimens. All of the blood smears were examined by two experienced microscopists (one was an expert from the Sanliurfa National Malaria Eradication Centre and other was a study investigator) blinded to each other’s results according to the WHO competency assessment protocol [20]. The blood smears were then independently reexamined by two experienced microscopists. A smear was considered to be negative when no parasites were detected in the total area, where 200 WBCs were observed either by experts from the Sanliurfa Malaria Eradication Centre or the study investigators as described previously [21]. Thick blood smears were used to calculate parasitaemia (parasites/microlitre of blood), as described by Zeyrek et al. [22].

### DNA extraction from filter paper

DNA was extracted from filter blots using the QIAmp DNA Blood Mini Kit (Qiagen, Germany) according to the manufacturer’s instructions.

### DNA extraction from thick blood smears

Before the extraction of DNA, the slides were cleaned to remove oil residues. Approximately 20 µL of Tris–EDTA (TE) buffer was put on the thick blood smear. Whatman filter paper was cut into strips and placed on the slide to absorb the buffer. The filter paper absorbed the blood from the thick blood smear, and was held by sterile forceps and put into a 1.5 mL centrifuge tube. Next, the DNA from the filter paper was extracted using the QIAmp DNA Blood Mini Kit (Qiagen, Germany) according to the manufacturer’s instructions.

### DNA amplification by nPCR

The nPCR amplification strategy was used for genotyping the 18S ssrRNA genes of *P. vivax*, *P. falciparum*, *Plasmodium ovale*, and *Plasmodium malariae*, in which specific primers were used, as described by Snounou et al. [23]. For the first amplification reaction, 1 µL of the template DNA extracted from the blood samples spotted onto filter papers was used (Nested 1), in which the fragment extended by rPLU1 and rPLU5 (Table 1) was amplified. Next, 1 µL of the product of the first amplification reaction was used as a template DNA for the secondary amplification reaction (Nested 2), in which the genus-specific (rPLU3-rPLU4) and species-specific (rVIV1-rVIV2, rFAL1-rFAL2, rMAL1-rMAL2, and rOVA1-rOVA2) primer pairs were used for each of the four separate reactions [23] (Table 1). The PCR assays were performed using a Gene Amp PCR System 97000 (PE applied biosystems).

**Table 1:** Schematic representation of the *Plasmodium* ssrRNA genes and nPCR protocol [13]

Species	PCR product	Primer	Sequence	Reaction
*Plasmodium genus*-specific	235 bp	rPLU3 rPLU4	TTTTTATAAGGATAACTACGGAAAAGCTGT TACCCGTCATAGCCATGTTAGGCCAATACC	Nested 2
*Plasmodium genus*-specific	1.6–1.7 kb	rPLU1 rPLU5	TCAAAGATTAAGCCATGCAAGTGA CCTGTTGTTGCCTTAAACTTC	Nested 1
*Plasmodium* species-specific				
*P. falciparum*	206 bp	rFAL1 rFAL2	TTAAACTGGTTTGGGAAAACCAAATATATT ACACAATGAACTCAATCATGACTACCCGTC	Nested 2
*P. malaria*	145 bp	rMAL1 rMAL2	ATAACATAGTTGTACGTTAAGAATAACCGC AAAATTCCCATGCATAAAAAATTATACAAA	Nested 2
*P. ovale*	226 bp	rOVA1 rOVA2	ATCTCTTTTGCTATTTTTTAGTATTGGAGA ATCTAAGAATTTCACCTCTGACATCTG	Nested 2
*P. vivax*	121 bp	rVIV1 rVIV2	CGCTTCTAGCTTAATCCACATAACTGATAC ACTTCCAAGCCGAAGCAAAGAAAGTCCTTA	Nested 2

All of the amplification reactions were carried out in a total volume of 20 µL and in the presence of 10 mM Tris–HCl, pH 8.3, 50 mM KCl, 250 nM of each oligonucleotide primers, 125 µM of each of the four deoxyribonucleotide triphosphates (dNTPs), 2.5 mM MgCl2, and 0.4 units of Taq DNA polymerase (Qiagen, Germany).

The cycling parameters for the PCR were as follows: step 1, initial denaturation at 95 °C for 5 min; step 2, annealing at X °C for 2 min (X = 58 °C for Nested 1, X = 64 °C for Nested 2); step 3, extension at 72 °C for 2 min; step 4, denaturation at 94 °C for 1 min; step 5, repeat steps 2–4 for a total of 25 cycles (Nested 1) or 30 cycles (Nested 2); step 6, final annealing at X °C for 2 min (X = 58 °C for Nested 1, X = 64 °C for Nested 2); step 7, final extension at 72 °C for 5 min; and step 8, reducing the temperature to 4 °C. The PCR products were stored at 4 °C until analysis.

The amplified products were electrophoresed on 2 % agarose gels performed in Tris–borate-EDTA for *P. vivax*, *P. falciparum*, *P. malariae*, and *P. ovale*, and stained with ethidium bromide for visual detection by ultraviolet transilluminator.

### Ethical approval

All procedures performed in this study involving human participants were in accordance with the ethical standards of the institutional and/or national research committee and with the 1964 Helsinki declaration and its later amendments or comparable ethical standards. This study was approved by the Ethics Committee of the Faculty of Medicine at Firat University (22.09.2011/13/13).

### Statistical analysis

Data entry and analyses were performed using Statistical Package for the Social Sciences 11.5. The descriptive data was given as a means with standard deviations, frequency counts, and percentages. Considered the gold standard in the diagnosis of malaria diagnostic methods based on microscopic diagnosis of malaria in the nPCR analysis of validity and reliability of the methodology for the determination of epidemiological research methods were used. nPCR analysis’ sensitivity, specificity, reliability, positive predictive value (PPV), negative predictive value (NPV) were analysed.

## Results

### Study area and population

The study sample comprised 153 suspected malaria patients, aged between 1 month and 77 years, from three provinces of Sanliurfa between 2008–2011 (Table 2) [24]. The mean age of the participants was 21.10 ± 16.10, of which 50.4 % were female and 49.6 % were male. Of the participants, 61.54 % had headaches, chills, fever, and sweats; 15.39 % had nausea, vomiting, weakness, and tiredness; and 23.07 % had fever and headaches. It was found that 38.2 % of the participants had been treated by chloroquine and primaquine during our survey, but 61.8 % had not used any drugs for malaria.

**Table 2:** The distribution of the malaria cases were according to the provinces of Sanliurfa [24]

Provinces	Years											Total
	2001	2002	2003	2004	2005	2006	2007	2008	2009	2010	2011	
Centre	41	44	36	23	5	2	1	18	1	0	0	173
Akcakale^a^	0	0	3	0	0	0	0	0^b^	0^b^	0^b^	1^b^	4
Birecik	30	43	21	19	0	0	3	0	0	0	0	116
Bozova	0	0	0	0	0	0	0	0	0	0	0	0
Ceylanpinar	346	176	50	17	0	1	1	0	0	0	0	591
Halfeti	0	0	0	0	0	0	0	0	0	0	0	0
Hilvan	7	4	4	2	2	5	0	0	0	0	0	24
Siverek^a^	529	902	634	397	375	234	49	9^b^	3^b^	4^b^	0^b^	3136
Suruç	4	5	6	4	0	2	0	0	0	0	0	21
Viransehir	159	70	29	18	8	1	5	0	0	0	0	290
Harran^a^	1	0	0	0	0	0	1	34^b^	5^b^	0^b^	0^b^	40
Toplam	*1117*	*1244*	*783*	*480*	*390*	*245*	*60*	*61*	*9*	*4*	*1*	*4394*

^a^Study provinces of this study

^b^Number of malaria cases in study provinces

**Fig. 1:**
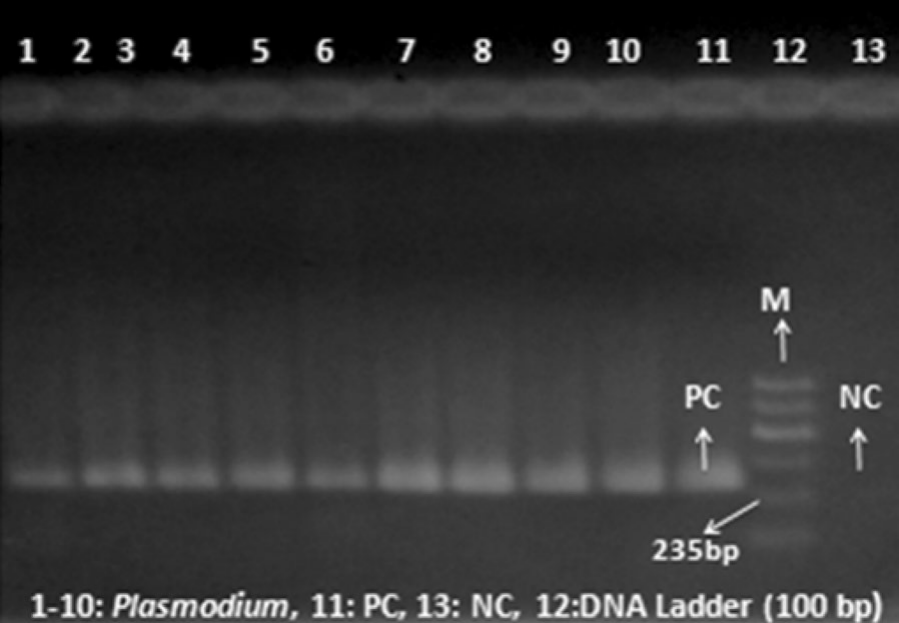
Agarose gel electrophoresis showing PCR products obtained using genus-specific primers; *Lanes* 1–10 *Plasmodium*, *Lane* 11 positive control, *Lane* 12 DNA marker, and *Lane 13* negative control

In the present study, 7.2 % (11) of the specimens were detected as *Plasmodium*-positive by microscopy. On the other hand, of all the 153 patients, 9.8 % (15) were determined as *Plasmodium*-positive using nPCR with *genus* specific primers (Fig. 1). The investigators were in doubt if the results of the nPCR were false-positive or not and the results of microscopical examination were false-negative or not. Two experienced microscopists examined the blood smears of the same patients twice and found that 7.2 % of the blood smears were still *Plasmodium*-positive and all of them were identified as only *P. vivax* by microscopic examination. According to the thin blood smears, the mean parasitaemia density was estimated as 4678.18 ± 3497.06 parasites/µL (range = 640–9760 parasites/µL) (Table 2). After twice repeating the nPCR, 15 patients were still *Plasmodium*-positive with nPCR. The investigators confirmed that all four specimens that were microscopically diagnosed as negative were determined as positive by nPCR. According to the results of the nPCR for malaria, these four patients were treated with chloroquine. After treatment with chloroquine, the symptoms of malaria in these suspected patients disappeared. Treatment and elimination of the malaria symptoms confirmed the accuracy of the nPCR that we found superior to microscopy. The WHO recommends that malaria should be confirmed by parasite-based diagnosis before giving treatment [25].

A total of 15 samples were diagnosed as *Plasmodium* and 93.33 % of them were *P. vivax* by nested PCR; three samples from Siverek, eight from Harran in 2008, three from Harran in 2009 and one from Akcakale in 2011. Although these samples were limited in small number size (n = 15), they were included in 26.8 % of total samples (n = 56) in study provinces: 43 in 2008, eight in 2009, four in 2010, one in 2011 (Table 2, number of malaria cases in study provinces). The patient diagnosed as *P. vivax* in Akcakale was the only case in 2011. The patient lived in a village which was located on the border with Syria. The patient’s house and Syrian houses were located face to face. The mean age of the patients infected with *P. vivax* was 25.92 ± 15.46 (range = 9–60 years), and 53.3 % were male.

**Fig. 2:**
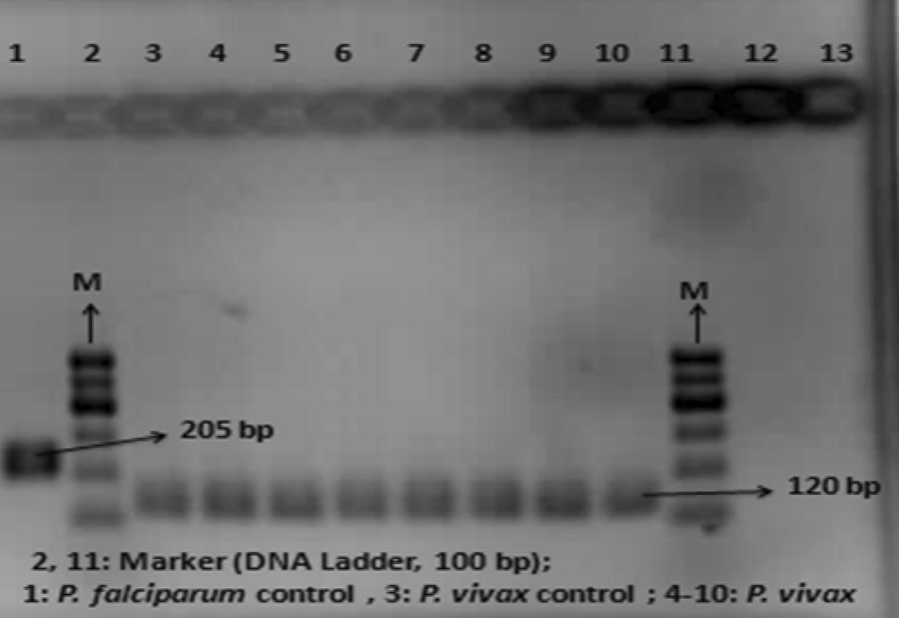
Agarose gel electrophoresis showing PCR products obtained using species-specific primers; *Lane* 1 *Plasmodium falciparum*-positive control, *Lane* 10 *Plasmodium vivax*-positive control, *Lanes* 2 and 11 Marker (DNA Ladder, 100 bp), *Lane* 12 negative control, *Lanes* 3–9 *Plasmodium vivax*

Of the *Plasmodium* specimens, 14 of 15 (93.33 %) were identified as *P. vivax* (Fig. 2) using species-specific primers (Table 1) for the identification of *P. vivax*, *P. falciparum*, *P. malaria*, *P. ovale*, respectively. One of the 15 *Plasmodium*-positive samples (Table 3, patient 3) was not identified with the species-specific primers used in this study.

**Table 3:** Results of the microscopic examination of *Plasmodium* and nPCR of *Plasmodium* and subspecies of *Plasmodium*

Number of cases	Microscopic examination	Number of parasitemia/µL	Diagnosis of *Plasmodium* by nPCR	Diagnosis of subspecies of *Plasmodium* by nPCR
1	Positive	640.00	*Plasmodium*	*Plasmodium vivax*
2	Positive	960.00	*Plasmodium*	*Plasmodium vivax*
3	Positive	1200.00	*Plasmodium*	–
4	Positive	1280.00	*Plasmodium*	*Plasmodium vivax*
5	Positive	1760.00	*Plasmodium*	*Plasmodium vivax*
6	Positive	5920.00	*Plasmodium*	*Plasmodium vivax*
7	Positive	6720.00	*Plasmodium*	*Plasmodium vivax*
8	Positive	7360.00	*Plasmodium*	*Plasmodium vivax*
9	Positive	7680.00	*Plasmodium*	*Plasmodium vivax*
10	Positive	8180.00	*Plasmodium*	*Plasmodium vivax*
11	Positive	9760.00	*Plasmodium*	*Plasmodium vivax*
12	Negative	–	*Plasmodium*	*Plasmodium vivax*
13	Negative	–	*Plasmodium*	*Plasmodium vivax*
14	Negative	–	*Plasmodium*	*Plasmodium vivax*
15	Negative	–	*Plasmodium*	*Plasmodium vivax*

Four out of the 15 specimens that were microscopically diagnosed as negative were positive for *Plasmodium* with nested-PCR (Table 4).

**Table 4:** Comparison of the microscopy and nPCR

	Microscopy					
	Positive		Negative		Total	
	n	%	n	%	n	%
nPCR						
Positive	11	73.3	4	26.7	15	*9.8*
Negative	0	0.0	138	100.0	138	90.2
Total	11	*7.2*	142	92.8	153	100.00

### Statistical analysis

When compared to the microscopy, the sensitivity, specificity, and positive predictive values of the nPCR were found as 100, 97.2 and 73.3 %, respectively.

## Discussion

The results of this study indicated that the examination of thick and thin blood smears by microscopy were insufficient for the diagnosis of malaria in this region. This study supported the idea that sensitivity decreases with microscopical tests as parasitaemia falls below 100 parasites/mL and false negatives are observed [2–6, 9, 26, 27]. When microscopy was used as the reference standard, it was found that the sensitivity, specificity, and positive predictive values of the nPCR as 100, 97.2, and 73.3 %, respectively. This study emphasized that nPCR was more sensitive and specific than microscopy, as it has been reported elsewhere [2, 6, 8, 26, 28–32].

Of the detected *Plasmodium*-positive samples, 93.33 % were detected as *P. vivax* and 6.77 % was not identified by targeting the 18S rRNA gene with the species-specific primers in the present study. According to the microscopical examination of the thick blood smear, it was identified as *P. vivax*. This result might be explained mostly in three ways: first, this patient was diagnosed by the technicians of the Sanliurfa National Malaria Eradication Centre, who only prepare thick and thin Giemsa-stained blood smears. They did not allow the adsorption of the patient’s blood onto the filter paper. They only sent the thick and thin blood smears to us for confirmation of the diagnosis of malaria. Hence, the investigators were obliged to extract DNA from the thick blood smears. This might be as a result of using DNA extraction from the thick blood smear. Because, most recently, Scopel et al. reported that the use of DNA extracted from thick blood smears resulted in poor detection of malaria parasites, particularly with parasite densities of less than 20/µL [33]. So, the most probable reason for amplification failure might be that the DNA became degraded or was of poor quality as it was obtained from a stained thick smear. Second, this might be due to their belonging to the newly determined fifth species of *Plasmodium.* When the patient was asked whether he had travelled to southeast Asia or not, it was seen that the patient had never travelled. So the idea of fifth species of *Plasmodium* is highly remote moreover the morphology of this parasite is quite distinct from that of *P. vivax*, which was confirmed microscopic diagnosis. Third, it might have been a case of the variant *P. ovale* as the oligonucleotide primer pairs used did not include those that can amplify this variant (Fig. 2).

## Conclusions

**Fig. 3:**
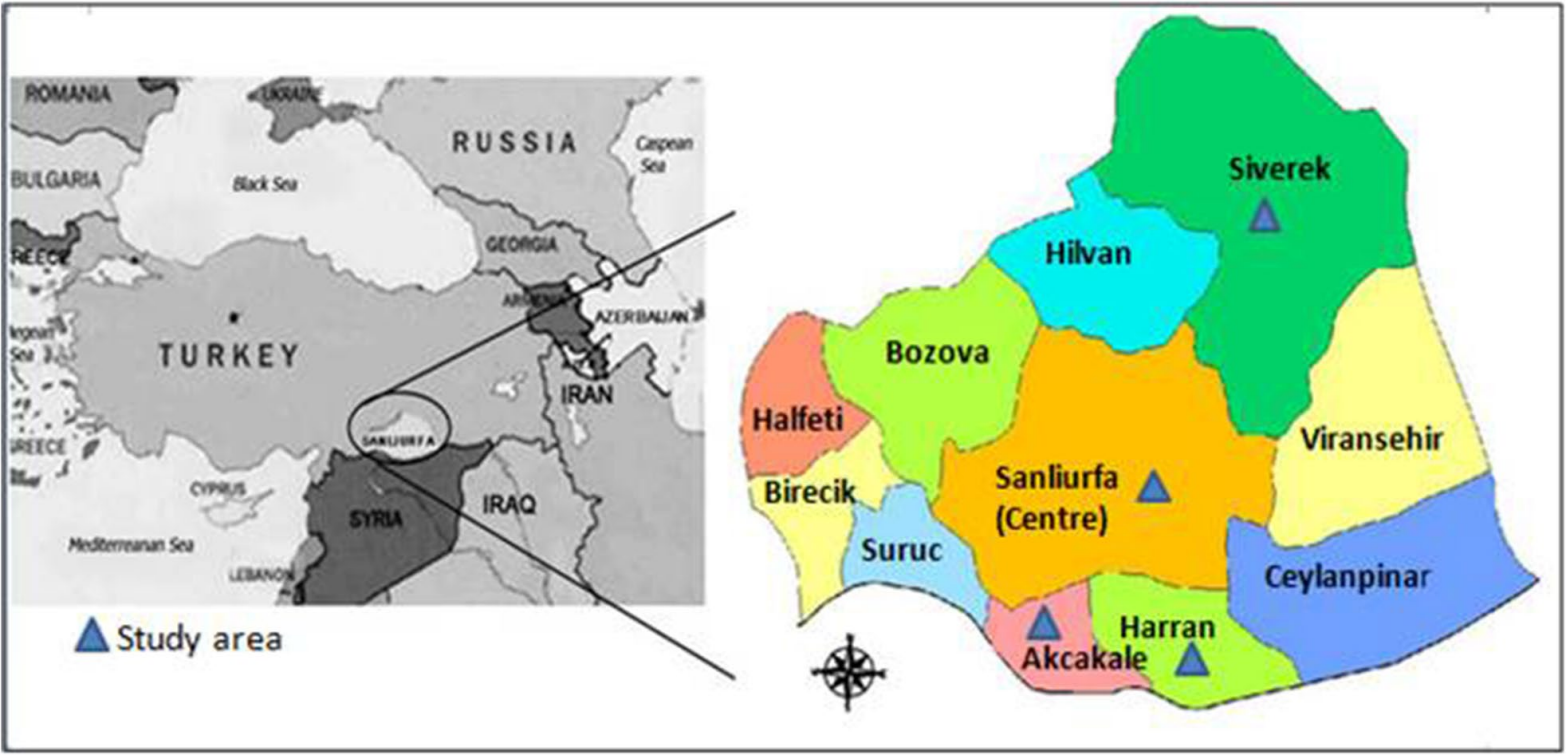
Study sites. Map of Sanliurfa showing the location of the study regions [22]

This study emphasized that nPCR is an excellent method for obtaining accurate epidemiological data in malaria endemic Sanliurfa (Fig. 3). The diagnosis of *Plasmodium* by nPCR might prevent misdiagnosis, incorrect treatment, false positives, false negatives, the emergence and spread of drug resistance, and the transmission of *Plasmodium* parasites from a malaria-endemic region to other provinces of Turkey. The nPCR was not affected by the subjectivity of the observers. In light of these data, nPCR might be a good and useful complement for clinically suspected but microscopically-negative malaria cases and epidemiological studies of *P. vivax* infections. nPCR might be a huge chance for the detection of low density of malaria parasites, mixed infections and identification asymptomatic carriers and reservoirs of parasites. nPCR in malaria suspected people might be a promising alternative to thick smear for screening for malaria in endemic region. Since, asymptomatic malaria carriers might be the parasite reservoir and responsible for the transmission of malaria in endemic region as it had previously been reported [34, 35]. The accurate diagnosis of *Plasmodium* and *Plasmodium* species, detection of *Plasmodium* DNA by nPCR from filter paper is easy to use and slightly invasive complement in malaria endemic region. As a result of the findings, nPCR is compulsory in Sanliurfa and it should be applied routinely in laboratory studies.

The results of the survey presented indicate that some cases of malaria might be missed by conventional microscopy and warrant the use of sensitive molecular techniques for surveillance; this should be placed in the current context where a large influx of displaced persons from neighbouring countries and some malaria-endemic countries are located in the GAP region. This raises a major concern that a potential re-introduction of malaria might occur in areas where it can rapidly disseminate into the local population. For instance, in the 2012 outbreak registered in the province of Mardin, a single index case of imported malaria with 218 autochthonous cases due to the passing of international truck drivers through intensive control and surveillance studies [36].
